# Supplementary figures and images for: Progesterone and prolactin levels in pregnant women living with HIV who delivered preterm and low birthweight infants: A nested case-control study
Source: PLoS One. 2023 Jan 23;18(1):e0280730. doi: 10.1371/journal.pone.0280730 (PMC9870101; doi:10.1371/journal.pone.0280730)

**S1 Fig: Histogram of progesterone concentrations across all participants**

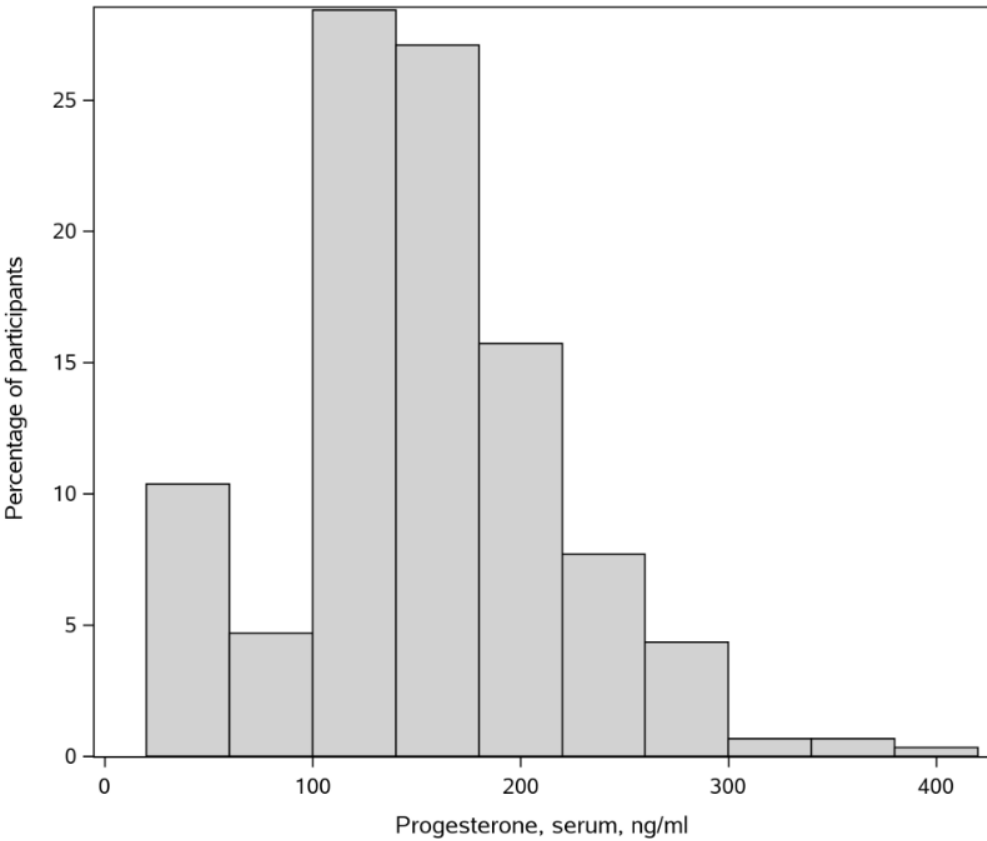

Supplement: S1 Fig — (PDF) [file pone.0280730.s001.pdf]

**S2 Fig: Histogram of prolactin concentrations across all participants**

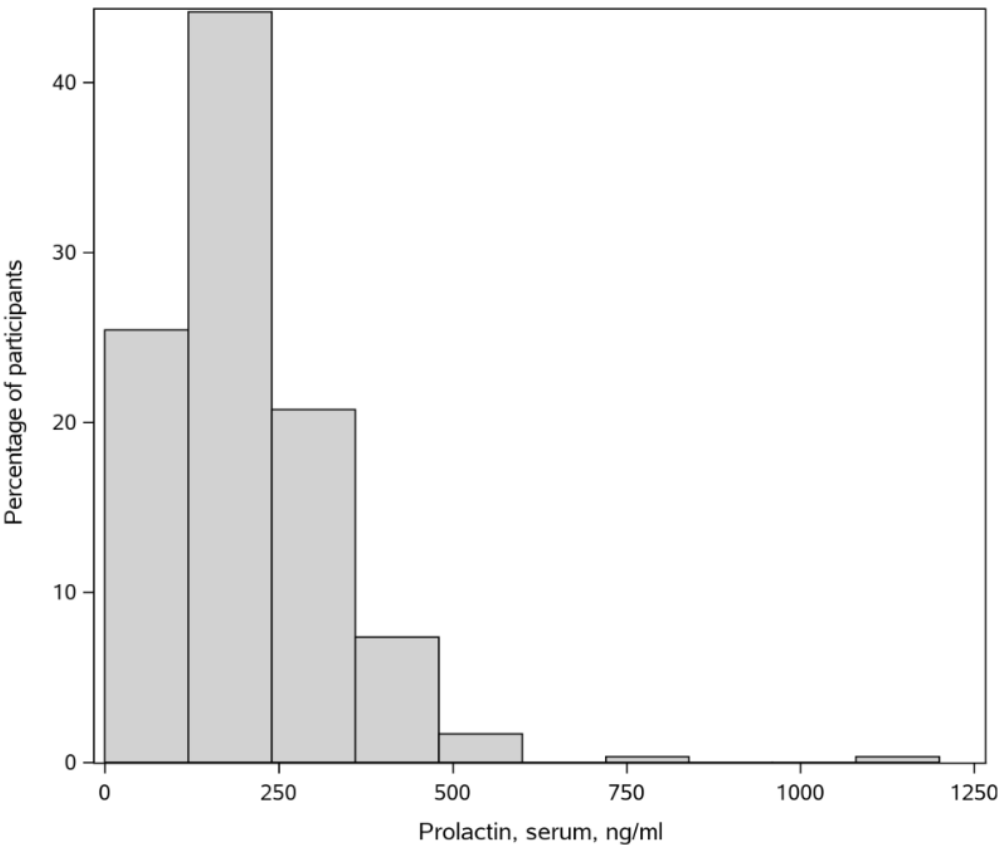

Supplement: S2 Fig — (PDF) [file pone.0280730.s002.pdf]
