# Supplementary material for: Progesterone and prolactin levels in pregnant women living with HIV who delivered preterm and low birthweight infants: A nested case-control study
Source: PLoS One. 2023 Jan 23;18(1):e0280730. doi: 10.1371/journal.pone.0280730 (PMC9870101; doi:10.1371/journal.pone.0280730)
Supplement: S1 Table — (PDF) [file pone.0280730.s003.pdf]

S1 Table: Relationship of hormone level and odds of preterm birth or low birth weight, stratified by antenatal antiretroviral regimen

	Threshold*	Adjusted** odds ratio (95%CI)	p value
**Antiretroviral therapy group**			
Progesterone	< 10th percentile	Ref	–
	≥ 10th percentile	1.62 (0.57, 4.56)	0.365
	< 25th percentile	Ref	–
	≥ 25th percentile	3.09 (1.44, 6.65)	0.004
Prolactin	< 10th percentile	Ref	–
	≥ 10th percentile	1.55 (0.56, 4.27)	0.396
	< 25th percentile	Ref	–
	≥ 25th percentile	1.36 (0.63, 2.94)	0.429
**Zidovudine only group**			
Progesterone	< 10th percentile	Ref	–
	≥ 10th percentile	2.49 (0.45, 13.8)	0.296
	< 25th percentile	Ref	–
	≥ 25th percentile	2.13 (0.75, 6.02)	0.156
Prolactin	< 10th percentile	Ref	–
	≥ 10th percentile	1.25 (0.26, 5.99)	0.778
	< 25th percentile	Ref	–
	≥ 25th percentile	0.91 (0.36, 2.32)	0.844

* Percentiles based on levels within corresponding gestational age strata. ** Adjusted models considered infant sex, country, and gestational age at start of antiretroviral regimen
