# Supplementary material for: Progesterone and prolactin levels in pregnant women living with HIV who delivered preterm and low birthweight infants: A nested case-control study
Source: PLoS One. 2023 Jan 23;18(1):e0280730. doi: 10.1371/journal.pone.0280730 (PMC9870101; doi:10.1371/journal.pone.0280730)

This is a well written report of a matched case-control study nested within the Antenatal Component of the PROMISE trial. The main hypothesis is that the observed difference observed in the parental trial between ZDV mono-therapy and ART could be explained by changes in progesterone and prolactin measured in the time window of 24-34 weeks of gestation. The fact that higher progesterone levels are associated with lower risk of (preterm birth (PTB) and/or low birthweight (LBW) is paradoxical so likely to be due to bias. Authors put the blame on misclassification of outcome and or exposure. Although, this cannot be ruled out, I have an alternative potential explanation for the findings. Unfortunately, because this alternative source of bias might have been introduced by a faulty study design, it cannot be removed but should be at least tested and discussed.

1. One of the key rules in case-control studies is that matching should be done only on potential confounders and not on mediators or colliders. There is no argument that age and country are potential confounders. However, it can be argued that gestational age is a proxy for the duration of exposure to treatment (this seems to be supported by data in Figure 1A showing that the higher the age the higher was the marker level). As such gestational age is a candidate for being a mediator in the relationship between treatment and outcome (see Figure, DAG below).
   Under this assumption, if there is an unmeasured factor U which is a cause of both gestational age and outcome, and analysis is adjusted for the mediator, this factor becomes a confounder for the association between progesterone levels and risk of outcome (as denoted by pink arrows in the graph). Failing to control for U will bias the association between progesterone levels and risk of outcome (potentially reversing the RR from <1, which is the expected one, to >1 which is the paradoxical finding). This hypothesis could be tested using simulated scenario for collision bias such as described here https://selection-bias.herokuapp.com/.

2. Still under the assumed DAG below, the key hypothesis lends itself to formally perform a mediation analysis as progesterone/prolactin are on the causal pathway from exposure to outcome. The authors correctly evaluate first the association between type of ART and progesterone/prolactin and separately the association between progesterone/prolactin levels and risk of PTB/LBW. However, the two analyses were not put together in a formal mediation analysis showing what percentage of the total effect of treatment on risk of outcome might be mediated by progesterone/prolactin levels. Showing that the total effect is similar to the direct effect would re-inforce the hypothesis that if there are indirect pathways they go through other unmeasured factors (e.g. estradiol) and not via changes in progesterone/prolactin. Because of the observational nature of the study (a cohort of the combined arms of Promise trial), analysis should be done using a counterfactual framework (e.g. Medeff command in Stata or equivalent).

3. Whatever the reason for the bias, I would emphasize even more in the Discussion section that i) paradoxical findings are often explained by bias (could mention other examples, e.g. obesity and survival conditioning on ICU) and ii) the fundamental problem of having introduced bias at the design stage (either because of

misclassification of outcome/exposure, or collider bias or both) that cannot be fixed at the analysis stage.

Minor point

First paragraph of the statistical analysis speaks about trends in progesterone/prolactin by gestational age. This could be interpreted as a longitudinal analysis with repeated measurements of progesterone/prolactin per participant, while it is cross-sectional. Suggest a rewording to clarify that is a simple correlation.

Figure

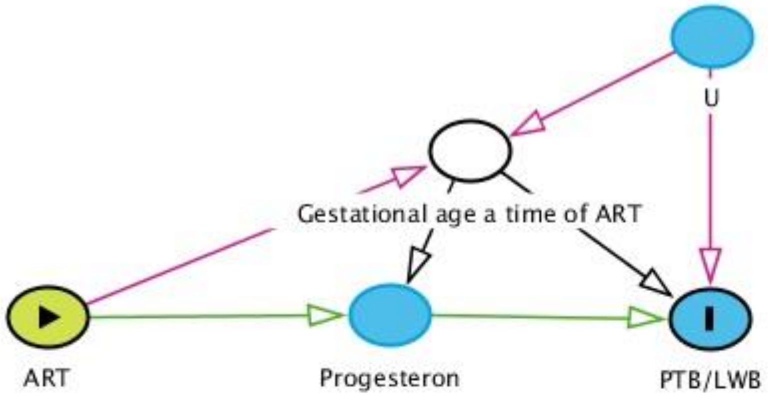

Supplement: Attachment — Submitted filename: Chi.pdf [file pone.0280730.s004.pdf]
