# Supplementary material for: Progesterone and prolactin levels in pregnant women living with HIV who delivered preterm and low birthweight infants: A nested case-control study
Source: PLoS One. 2023 Jan 23;18(1):e0280730. doi: 10.1371/journal.pone.0280730 (PMC9870101; doi:10.1371/journal.pone.0280730)
Supplement: Attachment — Submitted filename: Point-by-point responses.docx [file pone.0280730.s006.docx]

**Response to review comments**

**PONE-D-20-39706R1**

My biggest question was about the choice of a case control design and conditional logistic regression. Case-control designs are great in certain situations, but tend to have lots of potential biases. Plus, in my experiences, conditional logistic regression is limiting and has some quirks that sometimes lead to odd performance. I'm wondering if utilizing propensity scores or disease risk scores would be helpful. Since you have a continuous exposure, maybe using continuous propensity scores (<https://doi.org/10.1515/jci-2014-0022>) maybe incorporating stratification (<https://doi.org/10.1002/sim.8835>).

*We thank the reviewer for the question about the study design and suggestions. A case-control design was selected because we did not have sufficient funding to assay progesterone and prolactin on the over 3000 pregnant people who participated in the PROMISE study. In addition, we wanted to have a sufficient number of events to increase precision and allow the possibility of adjusting for a wide range of control variables. We agree that case-control designs have potential biases, but we believe that many such biases were limited in our study because the study was nested within the PROMISE study. This included matching variables and case status measured on participants who were not part of this specific analysis. Although we agree that conditional logistic regression—the standard analysis approach for case-control studies—has its drawbacks, this analysis strategy was sufficient and reliable enough to compute the odds ratios of interest in our study.*

*Nevertheless, we appreciate the suggestion of propensity scores as an alternative analysis strategy. The propensity score included the matching variables, but we used a discrete propensity model since the analysis strategy for the outcome model compared groups at different percentile levels. As can be seen in the two tables below, adjustment by propensity score tended to make the odd ratios more extreme.*

*This adjunct analysis reassures us about the direction of the results presented in the manuscript. As we do not have a specific reason why the propensity weighted analysis would be preferable than the conditional logistic regression, we prefer to use the results from conditional logistic regression, which appears to be more conservative.*

|  | | | | **Unadjusted** | | | | **Adjusted** | | | |  |
| --- | --- | --- | --- | --- | --- | --- | --- | --- | --- | --- | --- | --- |
| **Outcome Measure** | **Covariate** |  | **Level** | | **Odds Ratio (95% CI)** | **p-value** |  | | **Odds Ratio (95% CI)** | **p-value** |  | |
| PTB or LBW | progesterone |  | < 10th percentile | | Ref |  |  | | Ref |  |  | |
|  |  |  | ≥ 10th percentile | | 2.61 (1.84,3.70) | <.0001 |  | | 2.86 (1.84,4.44) | <.0001 |  | |
|  |  |  | < 25th percentile | | Ref |  |  | | Ref |  |  | |
|  |  |  | ≥ 25th percentile | | 1.68 (1.21,2.32) | 0.0019 |  | | 2.12 (1.43,3.13) | 0.0002 |  | |
|  | prolactin |  | < 10th percentile | | Ref |  |  | | Ref |  |  | |
|  |  |  | ≥ 10th percentile | | 1.60 (1.16,2.22) | 0.0045 |  | | 2.08 (1.35,3.22) | 0.0010 |  | |
|  |  |  | < 25th percentile | | Ref |  |  | | Ref |  |  | |
|  |  |  | ≥ 25th percentile | | 1.49 (1.08,2.06) | 0.0159 |  | | 1.53 (1.04,2.25) | 0.0291 |  | |

|  | | | | **Unadjusted** | | | | **Adjusted for Arm and Possible Confounders** | | | |  |
| --- | --- | --- | --- | --- | --- | --- | --- | --- | --- | --- | --- | --- |
| **Outcome Measure** | **Covariate** |  | **Level** | | **Odds Ratio (95% CI)** | **p-value** |  | | **Odds Ratio (95% CI)** | **p-value** |  | |
| PTB | progesterone |  | < 10th percentile | | Ref |  |  | | Ref |  |  | |
|  |  |  | ≥ 10th percentile | | 5.70 (3.64,8.92) | <.0001 |  | | 9.15 (5.15,16.3) | <.0001 |  | |
|  |  |  | < 25th percentile | | Ref |  |  | | Ref |  |  | |
|  |  |  | ≥ 25th percentile | | 2.12 (1.47,3.06) | <.0001 |  | | 3.08 (1.97,4.80) | <.0001 |  | |
|  | prolactin |  | < 10th percentile | | Ref |  |  | | Ref |  |  | |
|  |  |  | ≥ 10th percentile | | 1.84 (1.27,2.65) | 0.0012 |  | | 2.10 (1.31,3.35) | 0.0019 |  | |
|  |  |  | < 25th percentile | | Ref |  |  | | Ref |  |  | |
|  |  |  | ≥ 25th percentile | | 2.02 (1.40,2.91) | 0.0002 |  | | 1.98 (1.29,3.02) | 0.0016 |  | |

Specific comments:

1. (lines 122-129) I can understand why the timing of samples is important and appreciate that the authors have taken care to perform an adjustment. My issue here is that that the uncertainty introduced from the adjustment method is not incorporated into the modeling results. I consider what you've done similar to imputation with a single point and usually methods like multiple imputation are considered superior because they incorporate the uncertainty into the model. That said, I would think a Bayesian analysis would be superior here since the authors could place a distribution on the points that were converted. Based on the response to comment 2 from reviewer 1, the authors may have the experience to implement this.

*We thank the reviewer for the comment and the interesting perspective on the how we categorized progesterone and prolactin. While we agree that incorporating uncertainty in the cutoff used is important, the amount of additional uncertainty is minimal because there is uncertainty only in participants who have values around the cutoff. For example, a participant with a value in the 80^th^ percentile is very unlikely to, at a population level, have a value < 10^th^ or 25^th^ percentile. When incorporating uncertainty into the cut-offs, only a handful of participants change comparison groups. To see this directly, we utilized multiple imputation using quantile regression and a polynomial predictors for gestational age. We then imputed the population-level percentile cutoff and averaged the results using the standard multiple imputation combination rules. One challenge we encountered in implementing this strategy is that the category below the 10^th^ percentile contains so few participants that the imputation strategy often failed due to model instability. For this reason, we included the results for the 25^th^ percentile below. As can be seen in the results below, the results were similar due to the reasons noted above.*

*While these findings were informative, no changes were made to the manuscript. The results were similar and we were unable to provide reliable estimates for the analysis that compares groups cut at the 10^th^ percentile.*

|  | | | **Unadjusted** | | | | **Adjusted for Possible Confounders** | | | |
| --- | --- | --- | --- | --- | --- | --- | --- | --- | --- | --- |
| **Outcome Measure** |  | **ARV Arm** |  | **Odds Ratio (95% CI)** | **p-value** |  |  | **Odds Ratio (95% CI)** | **p-value** |  |
| progesterone |  | ZDV Only |  | Ref |  |  |  | Ref |  |  |
|  |  | ART Group |  | 1.99 (1.26,3.14) | 0.0033 |  |  | 1.71 (1.09,2.69) | 0.0200 |  |
|  |  |  |  |  |  |  |  |  |  |  |
| prolactin |  | ZDV Only |  | (Ref.) |  |  |  | (Ref.) |  |  |
|  |  | ART Group |  | 1.05 (0.68,1.63) | 0.8123 |  |  | 1.10 (0.67,1.80) | 0.7015 |  |

2. (line 133, lines 187-190, figure 1) Usually it's a strong assumption that a biomarker concentration increases linearly over time, though this may be true for progesterone and prolactin. I suggest including some information and justification on why this is a reasonable assumption in line 133. For lines 187-190, there are no confidence bands on the plots in figure 1 so it's not possible to tell if the lines really suggest a difference. In addition, I strongly encourage the authors to model these non-linearly since it's possible there's a nonlinear increase, especially for cases.

*We agree this can be a strong assumption. The linear line was originally intended as a visual aid, but we agree that it may be misleading. We have updated the manuscript to include new plots that utilize LOESS curves and 95% confidence intervals (as suggested).*

3. (lines 144-147) Given that the participants were ascertained as part of a randomized trial, I doubt the participants of the main trial represent any sort of meaningful group. Thus, it's not clear to me why weights are needed to "better replicate the full PROMISE study population" because "of the under-sampling of controls through our matching process." To me, that's an indication that matching is probably not the best approach to achieving a better estimate of the association.

*Thank you for the comment and perspective. While clinical trials are often criticized for a lack of external validity, we believe these concerns also depend on the study conducted. In this case, we have no reason to believe that the participants enrolled in PROMISE, with a population defined by the study entry criteria, would have a much different risk of an infant born premature or with a low birth weight in the general population of women living with HIV who become pregnant and seek antenatal care.*

*In our view, the weighted analysis is justified for several reasons. First, the parent trial has the advantage of a large sample size (over 3500 participants) across many sites in several regions. These features are unusual for a study of this nature and its findings are certainly more generalizable than smaller, single-site trials. Second, we observed higher probability of PTB-LBW in one arm (i.e., ART) over the other (i.e., antenatal ZDV). Without weights, we are concerned our findings would be biased due to conditioning on a future intercurrent event. Third, a common critique of observational studies is their lack of internal validity due to unmeasured confounding. By including selection weights to recover the original PROMISE population we retain internal validity of the randomized design. For these reasons, we believe that our efforts to increase external validity are worthwhile and serve to enhance—rather than limit—our findings.*

*Regarding the reviewer’s comment about matching: this approach has a long and established history in the context of case-control studies. Despite some limitations (including those acknowledge above), it is an accepted practice and a design feature that we are unable to change at this point.*

*We have not made any changes in the manuscript based on this comment. However, we are willing to add further justification in the paper itself if the reviewer considers this to be a major issue.*

4. (Table 1) Significance testing for baseline imbalance in randomized trials has been regarded as unnecessary (see Altman, https://doi.org/10.2307/2987510; Senn, https://doi.org/10.1002/sim.4780131703). This extends to observational analyses. I recommend removing the significance testing entirely from table 1. If an evaluation of difference is still desired, a standardized difference is better (see Austin, https://doi.org/10.1080/03610910902859574).

*Thank you for the comment. We have removed the p-values from the baseline table.*

5. (fig 1) If you can, please include the definitions for the points in the figure legend.

*Notations for cases (+) and controls (o) are noted in the caption.*

In addition, we have replaced reference #25 in the cited bibliography. The prior reference was a conference abstract. Since our last review, the full paper has been published. We are noting this in our response, as instructed.
