# Supplementary material for: Progesterone and prolactin levels in pregnant women living with HIV who delivered preterm and low birthweight infants: A nested case-control study
Source: PLoS One. 2023 Jan 23;18(1):e0280730. doi: 10.1371/journal.pone.0280730 (PMC9870101; doi:10.1371/journal.pone.0280730)
Supplement: Attachment — Submitted filename: FINAL response to reviewer comments v3.docx [file pone.0280730.s005.docx]

**REVIEWER 1**

This is a well written report of a matched case-control study nested within the Antenatal Component of the PROMISE trial. The main hypothesis is that the observed difference observed in the parental trial between ZDV mono-therapy and ART could be explained by changes in progesterone and prolactin measured in the time window of 24-34 weeks of gestation. The fact that higher progesterone levels are associated with lower risk of (preterm birth (PTB) and/or low birthweight (LBW) is paradoxical so likely to be due to bias. Authors put the blame on misclassification of outcome and or exposure. Although, this cannot be ruled out, I have an alternative potential explanation for the findings. Unfortunately, because this alternative source of bias might have been introduced by a faulty study design, it cannot be removed but should be at least tested and discussed.

1. One of the key rules in case-control studies is that matching should be done only on potential confounders and not on mediators or colliders. There is no argument that age and country are potential confounders. However, it can be argued that gestational age is a proxy for the duration of exposure to treatment (this seems to be supported by data in Figure 1A showing that the higher the age the higher was the marker level). As such gestational age is a candidate for being a mediator in the relationship between treatment and outcome (see Figure, DAG below).
Under this assumption, if there is an unmeasured factor U which is a cause of both gestational age and outcome, and analysis is adjusted for the mediator, this factor becomes a confounder for the association between progesterone levels and risk of outcome (as denoted by pink arrows in the graph). Failing to control for U will bias the association between progesterone levels and risk of outcome (potentially reversing the RR from <1, which is the expected one, to >1 which is the paradoxical finding). This hypothesis could be tested using simulated scenario for collision bias such as described here <https://selection-bias.herokuapp.com/>.

*We appreciate this comment and we agree that the careful selection of matching factors is essential. However, we respectfully disagree about the relationships described in the attached DAG. In our trial, women were randomized and started an antiretroviral regimen after screening for the study, implying that ART cannot be a cause of gestational age at the time of ART initiation. Because gestational age at the time of ART is a baseline factor in this randomized study, it seems more reasonable to place it as an upstream factor with no ART relationship. Furthermore, the time of initiation alone cannot serve as a reliable proxy for duration of the assigned regimen, since it is wholly dependent on the time of delivery, which as the parent PROMISE trial has shown varies by study arm. For these reasons, we do not agree with the proposed mediator relationship between “ART” and “gestational age at time of ART” and do not believe that this specific example of unmeasured confounding is valid in our trial scenario.*

*Having said that, we are in full agreement with the reviewer’s broader comment about unmeasured confounding. As with any study, unmeasured confounding can lead to unanticipated findings, including the seemingly paradoxical results we report here. Because this is a secondary analysis—and because the primary trial was not designed with this question in mind—we do not have the necessary data to rigorously conduct the hypothesis testing suggested above. We have included text in the Discussion that explicitly acknowledges this limitation (lines 296-299).*

2. Still under the assumed DAG below, the key hypothesis lends itself to formally perform a mediation analysis as progesterone/prolactin are on the causal pathway from exposure to outcome. The authors correctly evaluate first the association between type of ART and progesterone/prolactin and separately the association between progesterone/prolactin levels and risk of PTB/LBW. However, the two analyses were not put together in a formal mediation analysis showing what percentage of the total effect of treatment on risk of outcome might be mediated by progesterone/prolactin levels. Showing that the total effect is similar to the direct effect would re-inforce the hypothesis that if there are indirect pathways they go through other unmeasured factors (e.g. estradiol) and not via changes in progesterone/prolactin. Because of the observational nature of the study (a cohort of the combined arms of Promise trial), analysis should be done using a counterfactual framework (e.g. Medeff command in Stata or equivalent).

*We agree that a formal mediation analysis would strengthen our assertion in the Discussion about indirect pathways. Indeed, in an analysis using the procedures recommended above, we found that the progesterone levels contributed very little to the observed effect (see below).*

```
Causal Mediation Analysis
Quasi-Bayesian Confidence Intervals

                         Estimate 95% CI Lower 95% CI Upper p-value
ACME (control)            -0.0105      -0.0227         0.00   0.024 *
ACME (treated)            -0.0172      -0.0366         0.00   0.024 *
ADE (control)              0.1821       0.1305         0.23  <2e-16 ***
ADE (treated)              0.1754       0.1259         0.23  <2e-16 ***
Total Effect               0.1649       0.1120         0.22  <2e-16 ***
Prop. Mediated (control)  -0.0614      -0.1576        -0.01   0.024 *
Prop. Mediated (treated)  -0.1023      -0.2461        -0.01   0.024 *
ACME (average)            -0.0138      -0.0296         0.00   0.024 *
ADE (average)              0.1787       0.1279         0.23  <2e-16 ***
Prop. Mediated (average)  -0.0818      -0.2043        -0.01   0.024 *
---
Signif. codes:  0 '***' 0.001 '**' 0.01 '*' 0.05 '.' 0.1 ' ' 1
Sample Size Used: 298
```

*One highlight of the analysis is that the direct effect (0.1787) is larger than the total effect of the ART arm (0.1649). This is also why the signs for the mediated effect is negative (-0.0296). Stated another way, progesterone as mediator is “blunting” the effect of ART. One could say that, under the model, there is another pathway from ART to prematurity that is larger than the pathway through progesterone.*

*While we agree this type of analysis would strengthen the case for indirect pathways, after thorough discussions among team members, we ultimately decided not to include it formally in this paper. Such an analysis would require greater depth than is possible in the current report and we are exploring a possible separate manuscript to address these findings.*

*In addition, many factors may have led to our somewhat paradoxical findings, including misclassification, bias, and unmeasured confounding. Without a better accounting of those factors, we were concerned that inclusion of such a mediation analysis would place undue weight on our primary findings and that this could lead to misinterpretations of our results in the broader context.*

3. Whatever the reason for the bias, I would emphasize even more in the Discussion section that i) paradoxical findings are often explained by bias (could mention other examples, e.g. obesity and survival conditioning on ICU) and ii) the fundamental problem of having introduced bias at the design stage (either because of misclassification of outcome/exposure, or collider bias or both) that cannot be fixed at the analysis stage.

*We fully agree. We have included this as a potential explanation in the Discussion (lines 269-271).*

Minor point
First paragraph of the statistical analysis speaks about trends in progesterone/prolactin by gestational age. This could be interpreted as a longitudinal analysis with repeated measurements of progesterone/prolactin per participant, while it is cross-sectional. Suggest a rewording to clarify that is a simple correlation.

*We have edited the text to address this issue (lines 132-134): “For each participant, we measured progesterone and prolactin at a single time point. In our analysis, we described the distribution of these measurements, using linear regression to illustrate trends over time for the study population.”*

**REVIEWER 2**

This is an important study with great ramifications for the current global efforts toward eliminating mother to child transmission of HIV. Please find below comments for the authors to consider.

*Thank you for the positive comments. We address each of the comments individually below.*

Methods
1. Notwithstanding that the methods of the parent trial have been described extensively elsewhere, I recommend that the authors include relevant dates, including periods of recruitment for this study in this current article.

*The period of recruitment was included in the original manuscript (now line 169). This substudy did not recruit or enroll women separately. Instead, we analyzed stored specimens from participants who met eligibility criteria as cases and selected controls.*

Results
2. I recommend that the authors provide information relating to the numbers of participants potentially eligible, examined for eligibility, confirmed eligible, included in the study. Please give information separately for cases and controls.

*These details have been included in lines 169-174.*

3. I recommend that the authors provide important characteristics of study participants. For example, socio-demographic and clinical characteristics. Information on exposures and potential confounders should be provided.

*These data were included in Table 1 of the original submission. This included key maternal and newborn characteristics, compared by case and control groups.*

Discussion
4. In this manuscript, the information in the first paragraph of the discussion section should be under the introduction section. Specific objectives and any prespecified hypothesis including the basis for such hypothesis should be under the introduction. Ideally, the first paragraph of the discussion should describe the key findings/results of the study.

*This first paragraph has been revised to address these critiques. We still describe our central hypothesis, but in a way that provides context to the summary of key results that follow.*

5. I recommend that the authors should clearly discuss the generalisability (external validity) of the study results.

*We comment on the external validity of our findings in lines 292-296. While this study was only conducted at two of the PROMISE sites, the findings are likely generalizable to the full study population, given the standardized approach to clinical management and the biological nature of the primary exposures (i.e., progesterone, prolactin).*

6. For the findings of this study to be useful to clinicians, public health practitioners and policy makers, it is important that the authors provide in a separate section under discussion the practical implications of their findings for policy, practice and public health.

*This analysis explores the causal pathways between HIV, ART, and adverse birth outcomes. The underlying biology of these mechanisms is important and, if properly elucidated, could lead to important interventions to prevent preterm birth. However, such developments are downstream of the current research. At this stage, our somewhat paradoxical findings do not have many practical implications for policy and practice. Instead, we offer recommendations to other clinical and translational investigators about how these findings might shape future work.*

**REVIEWER 3**

The authors describe the results of a nested case control study of the PROMISE RCT evaluating pregnancy progesterone and prolactin levels in a subset of women with preterm and low birth weight infants appropriately matched controls. Mechanisms underlying adverse birth outcomes are poorly understood as such this manuscript seeks to contribute towards filling this gap in the literature. In this manuscript, the authors find that the ART exposure was associated with lower levels of progesterone, however contrary to their hypothesis PTB/LBW was associated with higher levels of progesterone. I would like to congratulate the authors on a thoughtful analysis, that is a valuable addition to the literature. I have the following minor comments:

*Thank you so much for the positive comments. We have addressed each of the critiques below.*

General

1. There is an interchange between the use of “women living with HIV” or “HIV-positive women” could the authors be consistent throughout

*For consistency, we have changed this to “HIV-positive women” throughout the body of the text.*

Introduction
2. Perhaps the authors could include some information on the normal changes in levels of progesterone and prolactin during normal pregnancy

*We agree with this suggestion but, given the flow of the paper, we found it difficult to include these details in the Introduction. However, we have included this detail in the Methods, along with supporting citations (lines 122-123).*

3. Linked to this - were specimens measured between 24-34 weeks because of study protocol (when specimens were collected) or because it was the most appropriate period physiologically?

*We originally targeted the early third trimester (i.e., 28 to 30 weeks gestation), reasoning that it would be late enough to be proximal to time of most deliveries while being early enough for intervention (if a relationship were to be found). Due to the distribution of specimens (which was timed from study entry rather than specific gestational ages), we expanded this window to 24 to 34 weeks. This is described in further detail in lines 122-128.*

Methods

4. “Briefly, pregnant women living with HIV…..were enrolled from 14 weeks gestation onwards.” Was gestational age determined at enrolment by LMP or SFH or only at delivery (using NBS)? Is enrolment GA data not available? Was there an upper limit for enrolment?

*At time of enrollment, gestational age was determined according to local obstetrical practices, typically measured by last menstrual period and physical exam. The earliest a participant could be enrolled was 14 weeks gestation; because of the limited availability of obstetrical ultrasound, however, most enrollments occurred later in the second trimester. There was no upper limit to enrollment.*

*While local practices were used to determine gestational age for eligibility, the gestational age used in our analyses was based on newborn examination. Therefore, the timing of enrollment and timing of specimen collection noted in our results are based on that back-calculation. This has been used across the different primary and secondary analyses of the PROMISE trial, and we use that same approach here for the sake of consistency and comparability.*

5. “we conducted a nested case-control study to investigate the associations between mid-trimester progesterone….” Is this mid trimester or mid pregnancy?

*Thank you for pointing this out. We agree with the characterization of “mid-pregnancy” and have made this change.*

6. “Delivery outcomes, including infant birth weight…” Was birthweight measured at delivery or within 5 days of life (as stated in Table 1)?

*We document weight at birth for those with institutional deliveries; however, some births occurred outside the facility. For those newborns, birth weights were collected as soon as possible afterwards (and within five days). To minimize confusion around this point, we have edited this sentence to read: “Delivery and early neonatal outcomes were collected on all infants born in the study” (line 97).*

7. “Participants were selected from two major enrolment sites.” Could the authors justify the inclusion of only two of the enrolment sites

*Because of funding constraints, we were only able to conduct the study in two sites. Both were already linked to the laboratory at Johns Hopkins that performed the progesterone and prolactin testing. We have revised the highlighted sentence to minimize confusion. Lines 104-106 now read: “This substudy analyzed stored specimens and clinical data from two major enrollment sites for the PROMISE study: Makerere University–Johns Hopkins University Research Collaboration (Kampala, Uganda) and College of Medicine-Johns Hopkins Research Project (Blantyre, Malawi).” We also note this as a possible limitation in lines 292-296.*

Discussion
8. “The New Ballard Score…..could lead to misclassification of cases and controls.” Could the authors speculate how misclassification could have lead to the unexpected results? Was this misclassification likely to be non-differential or differential?

*In secondary analysis of the PROMISE trial, when compared to the gold standard of obstetric ultrasound, we found that the performance of the New Ballard Score varied at different gestational age thresholds. If this misclassification were to be more likely among the cases or the controls (which seem plausible, since the groups were also determined according to gestational age, or proxies thereof), then it could affect our results (lines 258-262).*

**JOURNAL REQUIREMENTS**

1. Please ensure that your manuscript meets PLOS ONE's style requirements, including those for file naming. The PLOS ONE style templates can be found at

<https://journals.plos.org/plosone/s/file?id=wjVg/PLOSOne_formatting_sample_main_body.pdf>

and

<https://journals.plos.org/plosone/s/file?id=ba62/PLOSOne_formatting_sample_title_authors_affiliations.pdf>

*We have re-formatted the paper to comply with PLOS ONE style requirements.*

2. In your Methods section, please provide additional information about the participant recruitment method and the demographic details of your participants. Please ensure you have provided sufficient details to replicate the analyses such as: a) a description of how participants were recruited, and b) descriptions of where participants were recruited and where the research took place (hospital/site name).

*We have added details about how cases and controls were selected to be part of the study. We have also included the site names in the Methods to provide greater clarity as to where the participants were recruited.*

3. Please ensure you have included the registration number for the clinical trial referenced in the manuscript.

*These have been added in line 84.*

4. Please ensure you have discussed any potential limitations of your study in the Discussion, including study design, sample size and/or potential confounders.

*Although limitations had been discussed previously, we provide a clearer accounting of these issues in the revised manuscript. These can be found in lines 292-305.*

5. Please provide additional details regarding participant consent. In the ethics statement in the Methods and online submission information, please ensure that you have specified (1) whether consent was informed and (2) what type you obtained (for instance, written or verbal, and if verbal, how it was documented and witnessed). If your study included minors, state whether you obtained consent from parents or guardians. If the need for consent was waived by the ethics committee, please include this information.

*An ethics statement section has been included in lines 161-167 of the manuscript. This information has also been entered into the online submission portal.*

6. We note that you have indicated that data from this study are available upon request. PLOS only allows data to be available upon request if there are legal or ethical restrictions on sharing data publicly. For information on unacceptable data access restrictions, please see <http://journals.plos.org/plosone/s/data-availability#loc-unacceptable-data-access-restrictions>.

In your revised cover letter, please address the following prompts:

a) If there are ethical or legal restrictions on sharing a de-identified data set, please explain them in detail (e.g., data contain potentially identifying or sensitive patient information) and who has imposed them (e.g., an ethics committee). Please also provide contact information for a data access committee, ethics committee, or other institutional body to which data requests may be sent.

b) If there are no restrictions, please upload the minimal anonymized data set necessary to replicate your study findings as either Supporting Information files or to a stable, public repository and provide us with the relevant URLs, DOIs, or accession numbers. Please see <http://www.bmj.com/content/340/bmj.c181.long> for guidelines on how to de-identify and prepare clinical data for publication. For a list of acceptable repositories, please see <http://journals.plos.org/plosone/s/data-availability#loc-recommended-repositories>.

We will update your Data Availability statement on your behalf to reflect the information you provide.

*The IMPAACT network has established procedures related to data sharing, which are publicly available (*[*https://www.impaactnetwork.org/resources/manual-procedures*](https://www.impaactnetwork.org/resources/manual-procedures)*). As a study funded by this network, we are bound to these policies. Having said that, we have modified the language previously provided, so that it better explains the process. This text was used in another PROMISE analysis published in PLOS ONE in January 2020. As such, we hope it meets the journal’s requirements about this important issue:*

*“Due to ethical restrictions in the study’s informed consent documents and in the IMPAACT Network’s approved human subjects protection plan, study data are available upon request from* [*sdac.data@sdac.harvard.edu*](mailto:sdac.data@sdac.harvard.edu) *with the written agreement of the International Maternal Pediatric Adolescent AIDS Clinical Trials (IMPAACT) network. Data are also available to all interested researchers upon request to the IMPAACT Statistical and Data Management Center’s data access committee (email address:* [*sdac.data@fstrf.org*](mailto:sdac.data@fstrf.org)*); this committee reviews and responds to requests for data, obtains necessary approvals from IMPAACT leadership and the NIH, arranges for signature of a Data Use Agreement, and sends the requested data.”*

7. Thank you for stating the following in the Financial Disclosure section:

[Overall support for the International Maternal Pediatric Adolescent AIDS Clinical Trials Network (IMPAACT) was provided by the National Institute of Allergy and Infectious Diseases (NIAID) with co-funding from the Eunice Kennedy Shriver National Institute of Child Health and Human Development (NICHD) and the National Institute of Mental Health (NIMH), all components of the National Institutes of Health, under Award Numbers UM1AI068632 (IMPAACT LOC), UM1AI068616 (IMPAACT SDMC) and UM1AI106716 (IMPAACT LC), and by NICHD contract number HHSN275201800001I. Additional investigator support was provided by NIAID (BHC, K24AI120796) and the Fogarty International Center (JTP, K01TW010857). The study products in the PROMISE trial were provided free of charge by Abbott, Gilead Sciences, Boehringer Ingelheim, and GlaxoSmithKline. The content is solely the responsibility of the authors and does not necessarily represent the official views of the National Institutes of Health. For this substudy, the funders had no role in study design, data collection and analysis, decision to publish, or preparation of the manuscript.].

We note that you received funding from a commercial source: Abbott, Gilead Sciences, Boehringer Ingelheim, and GlaxoSmithKline

Please provide an amended Competing Interests Statement that explicitly states this commercial funder, along with any other [relevant declarations](about:blank) relating to employment, consultancy, patents, products in development, marketed products, etc.

Within this Competing Interests Statement, please confirm that this does not alter your adherence to all PLOS ONE policies on sharing data and materials by including the following statement: "This does not alter our adherence to PLOS ONE policies on sharing data and materials.” (as detailed online in our guide for authors [http://journals.plos.org/plosone/s/competing-interests](about:blank)). If there are restrictions on sharing of data and/or materials, please state these. Please note that we cannot proceed with consideration of your article until this information has been declared.

Please include your amended Competing Interests Statement within your cover letter. We will change the online submission form on your behalf.

Please know it is PLOS ONE policy for corresponding authors to declare, on behalf of all authors, all potential competing interests for the purposes of transparency. PLOS defines a competing interest as anything that interferes with, or could reasonably be perceived as interfering with, the full and objective presentation, peer review, editorial decision-making, or publication of research or non-research articles submitted to one of the journals. Competing interests can be financial or non-financial, professional, or personal. Competing interests can arise in relationship to an organization or another person. Please follow this link to our website for more details on competing interests: [http://journals.plos.org/plosone/s/competing-interests](about:blank)

*We have included text for Competing Interests Statement in the cover letter, which reads:*

*“The study products in the PROMISE trial were provided free of charge by Abbott, Gilead Sciences, Boehringer Ingelheim, and GlaxoSmithKline. This does not alter our adherence to PLOS ONE policies on sharing data and materials. Funders had no role in study design, data collection and analysis, decision to publish, or preparation of the manuscript. The authors have declared that no competing risks exist.”*

*If additional information is needed, please let us know.*

8. Please include captions for your Supporting Information files at the end of your manuscript, and update any in-text citations to match accordingly. Please see our Supporting Information guidelines for more information: [http://journals.plos.org/plosone/s/supporting-information](about:blank).

*This has been added according to PLOS ONE guidance.*
